# Supplementary material for: A UMLS-based spell checker for natural language processing in vaccine safety
Source: BMC Med Inform Decis Mak. 2007 Feb 12;7:3. doi: 10.1186/1472-6947-7-3 (PMC1805499; doi:10.1186/1472-6947-7-3)
Supplement: Additional file 13 — RAP application directory. Sets up directory for RAP (RDF application for PHP) source code [file 1472-6947-7-3-S13.gz › rap/doc/tutorial/usingNamedGraphs.htm]

Using RAP's Resource-Centric ResModel API


### RDF API for PHP V0.9.2

# Using RAP's RDF Dataset API

This turorial is part of the RAP - Rdf API for PHP documentation.

Daniel Westphal, Chris Bizer  
July 2005

## Table of Contents

1. Introduction
2. Usage Examples

## 1. Introduction

This section gives an introduction into the ideas behind Named Graphs  and RAP's RDF datset API.

### 1.1 Named Graphs

The Semantic Web can be seen as a collection of RDF graphs. The RDF recommendation explains the meaning of any one graph, and how to merge a set of graphs into one, but does not provide suitable mechanisms for talking about graphs or relations between graphs. But the ability to express metainformation about graphs is required for:

- Data syndication systems need to keep track of provenance information, and provenance chains.- Restricting information usage: Information providers might want to attach information about intellectual property rights or their privacy preferences to graphs in order to restrict the usage of published information.- RDF repository housekeeping: Triple stores need mechanisms to replace and update parts of their content.- Access control: A triple store may wish to allow fine-grain access control, which appears as metadata concerning the graphs in the store.- Signing RDF graphs: For signing RDF graphs it is necessary to keep the graph that has been signed distinct from the signature, and other etadata concerning the signing, which may be kept in a second graph.- Expressing propositional attitudes such as modalities and beliefs.- Scoping assertions and logic where logical relationships between graphs have to be captured.

RDF reification has well-known problems in addressing these use cases. To avoid these problems several authors propose quads, consisting of an RDF triple and a further URIref or blank node or ID. The proposals vary widely in the semantic of the fourth element, using it to refer to information sources, to model IDs or statement IDs or more generally to contexts. Named Graphs propose a general and simple variation on RDF, using sets of named RDF graphs.

A set of Named Graphs is a collection of RDF graphs. Each one is named with a URIref.

The name of a graph may occur either in the graph itself, in other graphs, or not at all. Graphs may share URIrefs but not blank nodes. Named Graphs can be seen as a reformulation of quads in which the fourth elements distinct syntactic and semantic properties are clearly distinguished, and the relationship to RDFs triples, abstract syntax and semantics is clearer.

Named Graphs has been adopted by W3C Data Access Working Group as data model for the SPARQL query language.

Further details about Named Graphs are found in:

- W3C Named Graphs Website - Jeremy Carroll, Chris Bizer, Pat Hayes, Patrick Stickler: Named Graphs, Provenance and Trust

### 1.2 RAP Dataset API

RAP's quad- and named graph-centric Dataset API is an implementation of DAWG's RDF dataset. It provides methods for parsing, manipulating and serializing RDF datasets and pure sets of Named Graphs. It also provides an implementation of the TriX syntax for exchanging RDF datasets. Datasets can be held in momory or be persisted in a relational database.

The basic idea of the Dataset API is to have a set of Named Graphs plus one additional unnamed default graph. The dataset can be manipulated by adding and removing entire Named Graphs, or by working with individual Quads.

**API Overview** 

The tables below give an overview about RAPs main interfaces and their most important methods. See PHPDoc  for details about all methods.

|  |  |
| --- | --- |
| **NamedGraph** | |
| A collection of RDF triples which is named by a URI. The NamedGraph object wraps RAP models, meaning that MemModel and DbModel can be reused. | |
| setGraphName(URI) | Sets the name of the graph. |
| getGraphName(URI) | Returns the URI of the named graph. |
| plus all other methods from statement-centric model API. | |

  

|  |  |
| --- | --- |
| **Dataset** | |
| An RDF datset is a collection of named RDF graphs plus one additional unnamed default graph. RDF datsets can be accessed and modified by adding and removing NamedGraph instances, by adding, removing and finding Quads (RDF triples with an additional graph name) and by adding triple to the background graph. RDF datasets can be serialized using TriX. | |
| addNamedGraph(namedgraph) | Adds a NamedGraph to the set. |
| addQuad(quad) | Adds a quad to the set of Named Graphs in the RDF dataset. A new NamedGraph will automatically be created if necessary. |
| addDefaultGraph(graph) | Adds the default graph to the RDF dataset. |
| createGraph(graphName,baseURI) | Creates a new NamedGraph and adds it to the set. An existing graph with the same name will be replaced. |
| getNamedGraph(graphName) | Returns the NamedGraph with a specific name from the Dataset. |
| getDefaultGraph() | Returns a reference to the default graph of the Dataset. |
| removeNamedGraph(graphName) | Removes a NamedGraph from the set. |
| findInNamedGraphs(quad) | Finds Quads that match a quad pattern. |
| listNamedGraphs() | Returns an iterator over all NamedGraphs in the set. |
| serializeToFile() | Saves RDF datset as TriX file. |

  

## Usage Examples

### 2.1 Operations on Datasets and NamedGraphs

<?php   
// Include RAP   
define("RDFAPI\_INCLUDE\_DIR", "./../api/");   
include(RDFAPI\_INCLUDE\_DIR . "RdfAPI.php");

// Get a GraphSet from the ModelFactory   
$graphset = ModelFactory::getDatasetMem('Dataset1');

// Create a new NamedGraphsMem   
$graph1 = new NamedGraphMem('http://graph1');   
$graph2 =& $graphset->createGraph('http://graph2');

// Create Quads   
$quad1= new Quad(new Resource('http://graph3'),new Resource('http://subject1'),new Resource('http://predicate1'),new Resource('http://object1'));   
$quad2= new Quad(new Resource('http://graph3'),new Resource('http://subject2'),new Resource('http://predicate2'),new Resource('http://object2'));   
$quad3= new Quad(new Resource('http://graph4'),new Resource('http://subject3'),new Resource('http://predicate3'),new Resource('http://object3'));   
$quad5= new Quad(new Resource('http://graph4'),new Resource('http://subject5'),new Resource('http://predicate5'),new Resource('http://object5'));

// Add Quads to the GraphSet.   
//
The necessary NamedGraphs will be created automatically.   
$graphset->addQuad($quad1);   
$graphset->addQuad($quad3);   
$graphset->addQuad($quad2);

// Add a NamedGraph to the Set   
$graphset->addNamedGraph('http://graph1',$graph1);

// Is the Graph "http://graph1" part of the GraphSet?   
echo '<b>Is the Graph "http://graph1" part of the GraphSet?</b><BR>';   
var\_dump($graphset->containsNamedGraph('http://graph1'));   
echo'<p/>';

// Remove 'http://graph2' from the set   
$graphset->removeNamedGraph('http://graph2');

// Is the Graph "http://graph2" part of the GraphSet?   
echo '<b>Is the Graph "http://graph2" part of the GraphSet?</b><BR>';   
var\_dump($graphset->containsNamedGraph('http://graph2'));   
echo'<p/>';

// Create basic statement   
$statement=new Statement(new Resource('http://subject4'),new Resource('http://predicate4'),new Resource('http://object4'));

// Get a Graph from the Set and add a basic statement   
$graph4=&$graphset->getNamedGraph('http://graph4');   
$graph4->add($statement);

// Remove Quad5   
$graphset->removeQuad($quad5);

// Get an iterator over all Quads   
echo '<p/><b>All Quads:</b><BR>';   
for($iterator = $graphset->findInNamedGraphs(null,null,null,null); $iterator->valid(); $iterator->next())   
{   
   $quad=$iterator->current();   
   echo $quad->toString().'<BR>';   
};

// Get an iterator over all Quads in 'http://graph3'   
echo '<p/><b>All Quads in http://graph3:</b><BR>';   
for($iterator = $graphset->findInNamedGraphs(new resource('http://graph3'),null,null,null); $iterator->valid(); $iterator->next())   
{   
   $quad=$iterator->current();   
   echo $quad->toString().'<BR>';   
};

// How many Quads are in the whole GraphSet   
echo '<p/><b>How many Quads are in the whole GraphSet?</b><BR>';   
echo $graphset->countQuads();

// Does the GraphSet contain the Quad (http://graph4, http://subject4, http://predicate4, http://object4) ?   
echo '<p/><b>Does the GraphSet contain the Quad (http://graph4, http://subject4, http://predicate4, http://object4) ?</b><BR>';   
var\_dump($graphset->containsQuad(new Resource('http://graph4'),new Resource('http://subject4'),new Resource('http://predicate4'),new Resource('http://object4')));   
?>

### 2.2 Using Database Persistence

<?php  
// Include RAP  
define("RDFAPI\_INCLUDE\_DIR", "./../api/");  
include(RDFAPI\_INCLUDE\_DIR . "RdfAPI.php");  
  
// Get a DbStore from the modelFactory  
$dbstore=ModelFactory::getDbStore('MySql','localhost','rap','root','');  
  
// Create the necessary tables in the database, if they aren't already set up.   
if (!$dbstore->isSetup('MySQL'))  
   $dbstore->createTables('MySql');  
  
// Create a new dataset, if the dataset was not found   
$dataset1=& $dbstore->getDatasetDb('Dataset1');  
if ($dataset1 === false)  
   $dataset1=& $dbstore->getNewDatasetDb(''Dataset1'');  
  
// Create a new namedGraph in the dataset.   
$namedGraph1=& $dataset1->createGraph('http://rap/NamedGraph1');  
  
// Add a statement to the namedGraph
  
$namedGraph1->add(new Statement(new Resource('http://rap/ng1\_res1'),new Resource('http://rap/ng1\_res2'),new Resource('http://rap/ng1\_res3')));

// Create a Quad  
$quad1= new Quad(new Resource('http://rap/NamedGraph2'),new Resource('http://rap/ng2\_res1'),new Resource('http://rap/ng2\_res2'),new Resource('http://rap/ng2\_res3'));

// Add the Quad to the dataset (the needed namedGraph (http://rap/NamedGraph2) is created automatically)  
$dataset1->addQuad($quad1);

// Print all quads   
for($iterator = $dataset1->findInNamedGraphs(null,null,null,null); $iterator->valid(); $iterator->next())   
{  
   $currentQuad= $iterator->current();  
   echo $currentQuad->toString().'<BR>';  
};   
?>
